# *Ab initio* investigation of nonlinear optical, electronic, and thermodynamic properties of BEDT-TTF molecule: doping with boron

**DOI:** 10.1016/j.heliyon.2021.e07461

**Published:** 2021-07-03

**Authors:** G.F. Olinga Mbala, M.T. Ottou Abe, Z. Ntieche, G.W. Ejuh, J.M.B. Ndjaka

**Affiliations:** a Faculty of Science, Department of Physics, University of Yaoundé I, P.M.B 812, Yaoundé, Cameroon; b Department of Electrical and Electronic Engineering, National Higher Polytechnic Institute, University of Bamenda, P. O. Box 39, Bambili, Cameroon; c Department of General and Scientific Studies, IUT-FV Bandjoun, University of Dschang, P.M.B 134, Bandjoun, Cameroon; d Laboratory of Pharmaceutical Technology, Institute of Medical Research and Medicinal, Plants Studies, P.O Box 6163, Yaounde, Cameroon; e Local Materials Promotion Authority (MIPROMALO), P.O. Box 2396, Yaounde, Cameroon

**Keywords:** BEDT-TTF, Hyperpolarizability, Nonlinear optical properties, Doping, Optoelectronic, NBO

## Abstract

In this study, the RHF, B3LYP and wB97XD methods with cc-pVDZ basis set have been used to investigate the influence of carbon atoms substitution with boron atoms on the non-linear optical, electronic, optoelectronic and thermodynamic properties of BEDT-TTF ($C_{10}H_{8}S_{8}$). The results show that the undoped molecule denoted BEDT-TTF or ET (Eg = 3.88 eV) and its derivatives are semi-conductors materials. However, doping $C_{10}H_{8}S_{8}$ with both 3B and 2B, creating a strong donor-acceptor system and considerably improves its energies gap (Egap). The Eg values of these doped molecules are between 2.2 and 2.39 eV less than 3 eV, which makes more interesting electronic properties. The nonlinear optical parameters such as dipole moment (μ), average polarizability ˂α˃ and first-order hyperpolarizability ($\beta_{mol}$) have been calculated and compared with the corresponding values of Urea used as prototypical material to study the NLO properties of the compound. These values obtained indicate that these materials exhibit good nonlinear optical properties. Moreover, we have also computed the chemical softness (ς), ionization potential (IP), electron affinity (AE), global hardness (η), refractive index (n), dielectric constant (ε), electric field (E) and electric susceptibility (χ), total electronic energy (Eo), enthalpy H, entropy S. These results indicate that these new materials doped with boron are promising candidates for the construction of optoelectronics and photonic devices.

## 1 Introduction

**Figure 1:**
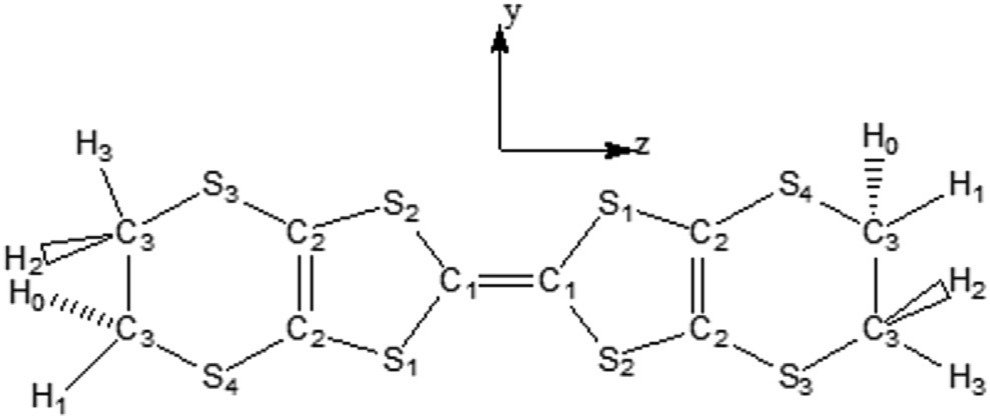
Molecular structure of BEDT-TTF.

Organic compounds represent promising materials, certainly, because they could find applications for example in modern communication, optical computing, dynamic image processing, telecommunication, data storage also in light-emitting diode (LED), field-effect transistor (FET) and other laser devices [1]. Organic semiconductors are generally pi-conjugated materials, in which the transport mechanisms are quite different from those conventionally described in solid-state physics with inorganic semiconductors [1]. With the discovery, of bis (ethylenedithio) tetrathiafulvalene abbreviated as BEDT-TTF or simply ET in 1980 [2] (Figure 1). In the literature, others organic conductors have been synthesized and studied previously [3]. Demiralp et al [4], has been reported that, about 30 organic superconductors based on BEDT-TTF have been synthesized with critical temperature up to 12,8K .In the previous study, the infrared and Raman spectra of BEDT-TTF have been reported by Kozlov et al [5], Eldridge et al [6] and Ruifeng et al [7]. Moreover, the works of Flakina et al [8], revealed new molecules from the monosubstituted anion of isocyanuric acid on BEDT-TTF and their crystal structures were determined. The chirality of substituted BEDT-TTF derivatives was in the heart of work of Wallis et al [9]. In addition, gives opportunities for preparing multifunctional materials. Much attention continues to be directed to the development of magnetic molecular conductors taking into account interactions between conductive electrons and localized spins. In this context, a global study of the vibrational dynamics of the bis(ethylenedithio) tetrathiafulvalene has been performed through *ab-initio* investigations [10, 11, 12, 13, 14]. These studies have contributed much to our knowledge of the vibrational spectra of these molecules. However, the nonlinear optical, electronic, optoelectronic and thermodynamics properties are still not fully understood. In recent years, many works [15, 16, 17, 18] have shown that the ab -initio and the density functional theory methods have become powerful tools in the study of electronic structure, optoelectronic and nonlinear optical properties. The goal of this original research is to propose materials, which find their applications in organic electronic. In this paper, we investigate the semi-conductor nature of the molecule bis (ethylenedithio) tetrathiafulvalene and some of its derivatives by doping it with some atoms, which may have application in quantum dots and other material. Our objectives are: to use *ab initio* and DFT quantum mechanical calculations to decipher the electronic structure, dipole moments, average polarizability, hyperpolarizabilities, first molecular hyperpolarizabilities, HOMO-LUMO molecular orbital diagram, dielectric constant, refractive index, energy gap, susceptibility, electrical conductivity, Electron affinity, Ionization potential and Molar refractivity of bis(ethylenedithio)tetrathiafulvalene with their doped-molecules. This work present the ab-initio and DFT calculations in the ground state using Hartree-Fock (HF), B3LYP (Becke-3-Lee-Yang-Parr) and wB97XD methods with a cc-pVDZ basis set.

We organized this paper in four sections. We present in section 2, the calculation method. In section 3, the results and discussion are presented. The conclusion is given in section 4.

## 2 Methods of calculation

The calculations were computed using Gaussian 09 calculation code [19]. To study the electronic structure of bis (ethylenedithio) tetrafulvalene (C_10_H_8_S_8_), and 3B-, 2B-doped BEDT-TTF ($C_{7}B_{3}H_{5}S_{8}$) and($C_{8}B_{2}H_{6}S_{8}$) respectively we first of all constructed these molecules by using Gauss view 6.0.16 modelling software [20]. Initially, pre-optimization was used employing the RHF level of theory with a minimal basis set. Then, the calculations were carried out using the polarized valence double zeta. DFT method was employed with Becke's three parameters non-local exchange functional with the Lee-Yang-Parr correlation function (B3LYP) functional [21, 22]. This functional is used because of their cost-effective method for the inclusion of electron correlations. To take into account the empirical dispersion and long-range correction, wB97XD functional has been used. The geometry optimization has been studied in the ground state.

## 3 Results and discussion

### 3.1 Optimized structure and geometric properties

#### 3.1.1 Optimized structure

**Figure 2:**
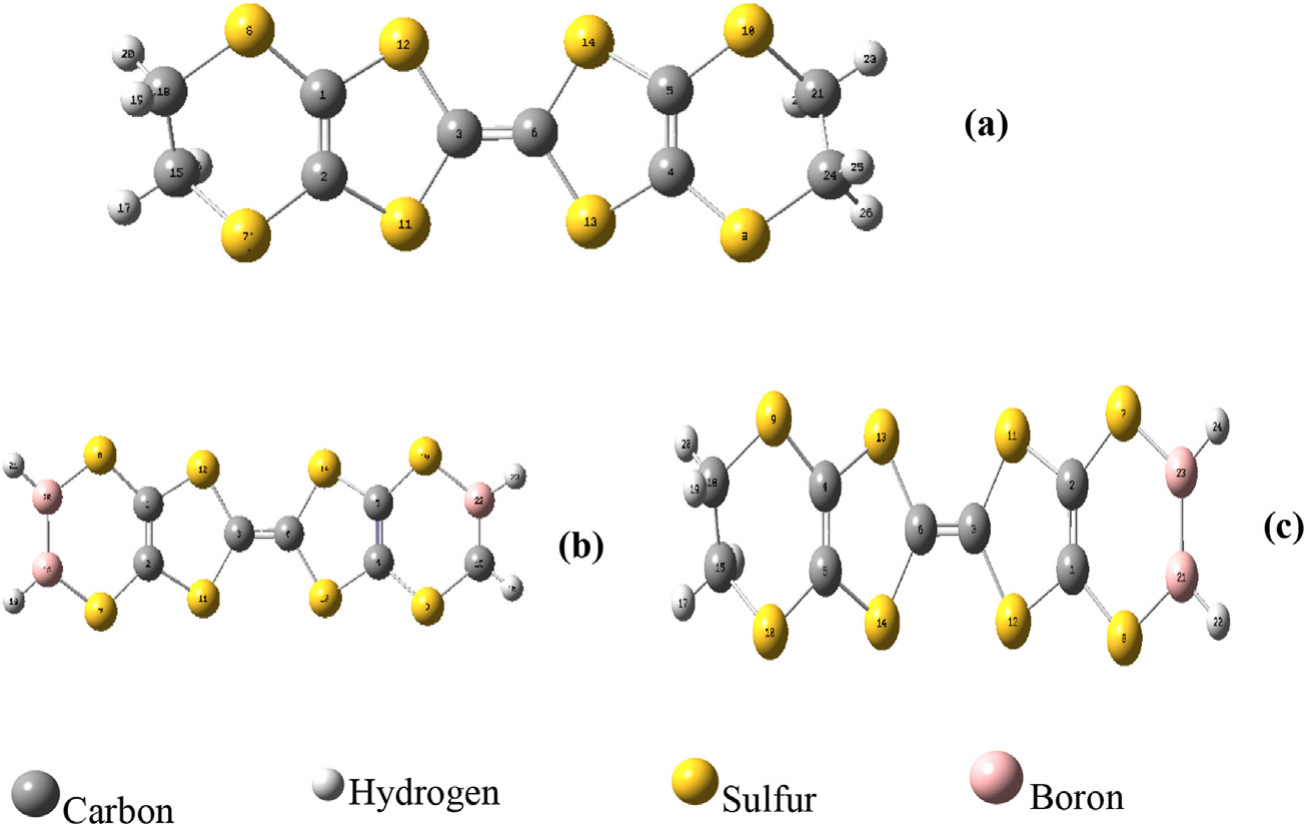
Optimized molecules of (a) bis (ethylenedithio) tetrathiafulvalene (ET), (b) 3B-doped (B3ET) and (c) 2B-doped to bromine (B2ET) with B3LYP/cc-pVDZ basis set.

Figure 2 shows the optimized molecules of bis (ethylenedithio) tetrafulvalene, undoped structure **(a)** and their derivatives **(b)** and **(c)** respectively $C_{7}B_{3}H_{5}S_{8}$ and $C_{8}B_{2}H_{6}S_{8}$, doped molecules. The geometric parameters of the neutral structure of BEDT-TTF are summarized in Table 1.

**Table 1:** Optimized geometric parameters of bis (ethylenedithio) tetrathiafulvalene (BEDT-TTF), bond lengths (Å) and angles (^o^) compared with experimentobtained with B3LYP, wB97XD and RHF using cc-pVDZ basis set.

Bond lengths(Å)	B3LYP	wB97XD	RHF	Exp. [23]
R(1,2)	1.3531	1.3467	1.3263	1.347
R(1,8)	1.7672	1.7596	1.772	1.745
R(1,12)	1.7885	1.7758	1.7816	1.768
R(2,7)	1.7672	1.7598	1.7706	1.746
R(2,11)	1.7877	1.775	1.7791	1.768
R(3,6)	1.3542	1.3472	1.33	1.352
R(3,11)	1.7843	1.7736	1.7756	1.762
R(3,12)	1.7861	1.7751	1.7766	1.770
R(4,5)	1.3531	1.3467	1.3263	1.350
R(4,9)	1.7671	1.7596	1.772	1.755
R(4,13)	1.7885	1.7758	1.7815	1.778
R(5,10)	1.7671	1.7598	1.7706	1.757
R(5,14)	1.7877	1.775	1.7791	1.771
R(6,13)	1.7861	1.7751	1.7765	1.763
R(6,14)	1.7843	1.7736	1.7756	1.758
R(7,15)	1.8477	1.8303	1.8175	1.835
R(8,18)	1.8672	1.8484	1.8255	1.824
R(9,24)	1.8674	1.8484	1.8254	1.811
R(10,21)	1.8478	1.8304	1.8177	1.817
R(15,16)	1.0996	1.0992	1.0886	-
R(15,17)	1.1014	1.1	1.0903	-
R(15,18)	1.5196	1.5176	1.5213	1.517
R(18,19)	1.0991	1.0984	1.0876	-
R(18,20)	1.1024	1.1009	1.0907	-
R(21,22)	1.0996	1.0992	1.0886	-
R(21,23)	1.1014	1.1	1.0903	-
R(21,24)	1.5196	1.5176	1.5213	1.518
R(24,25)	1.0991	1.0984	1.0876	-
R(24,26)	1.1024	1.1009	1.0907	-
**Bond angles (°)**				
A (2, 1,8)	127.4013	127.3211	129.3176	129.74
A (2, 1,12)	116.8399	116.9136	116.9503	116.91
A (8, 1,12)	115.7136	115.722	113.6787	113.01
A (1, 2,7)	123.5173	123.4119	127.4358	127.73
A (1, 2,11)	117.1404	117.2423	117.4037	116.77
A (7, 2,11)	118.7068	118.7058	115.1114	115.22
A (6, 3,11)	123.7415	123.5804	123.96	122.88
A (6, 3,12)	123.2783	123.1584	123.5578	123.01
A (11, 3,12)	112.9329	113.2254	112.4472	113.99
A (5, 4,9)	127.3958	127.321	129.3183	122.49
A (5, 4,13)	116.8399	116.9137	116.9533	117.49
A (9, 4,13)	115.7189	115.7221	113.6748	119.64
A (4, 5,10)	123.5121	123.4119	127.4371	125.08
A (4, 5,14)	117.139	117.2423	117.4039	117.06
A (10, 5,14)	118.71	118.7059	115.1101	117.83
A (3, 6,13)	123.277	123.1584	123.5563	122.88
A (3, 6,14)	123.7419	123.5803	123.9582	122.65
A(13,6,14)	112.9347	113.2254	112.4501	114.29
A(2,7,15)	96.1788	95.851	99.091	99.75
A(1,8,18)	103.7839	103.7149	102.5825	100.70
A(4,9,24)	103.7831	103.7149	102.58	96.61
A(5,10,21)	96.1814	95.851	99.089	103.49
A(2,11,3)	93.5086	93.3924	94.2293	94.51
A(1,12,3)	93.621	93.525	94.2803	94.05
A(4,13,6)	93.6219	93.525	94.2844	93.47
A(5,14,6)	93.5118	93.3924	94.2332	93.92
A (7,15,16)	107.5626	107.7423	109.568	-
A(7,15,17)	105.823	106.2662	104.7873	-
A(7,15,18)	113.0628	112.9638	112.3925	112.34
A(16,15,17)	108.6664	108.5855	108.6259	-
A(16,15,18)	112.0228	111.8243	111.5666	-
A(17,15,18)	109.4364	109.2284	109.6251	-
A(8,18,15)	115.7202	115.6584	114.054	113.38
A(8,18,19)	105.6108	105.9438	109.1802	-
A(8,18,20)	105.8332	106.1314	104.0093	-
A(15,18,19)	110.5685	110.2745	110.4982	-
A(15,18,20)	109.9151	109.7127	110.2224	-
A(19,18,20)	108.8816	108.8445	108.5828	-
A(10,21,22)	107.5579	107.7423	109.5662	-
A(10,21,23)	105.8207	106.2662	104.7844	-
A(10,21,24)	113.0656	112.9638	112.3834	117.19
A(22,21,23)	108.666	108.5855	108.631	-
A(22,21,24)	112.0235	111.8243	111.5713	-
A(23,21,24)	109.4399	109.2284-	109.6291	-
A(9,24,21)	115.7144	115.6584	114.0508	115.01
A(9,24,25)	105.6021	105.9438	109.1854	-
A(9,24,26)	105.835	106.1314	104.0089	-
A(21,24,25)	110.5733	110.2745	110.4964	-
A(21,24,26)	109.9181	109.7127	110.2242	-
A(25,24,26)	108.8866	108.8445	108.5813	-

#### 3.1.2 Geometric properties

The optimized geometric parameters of bis (ethylenedithio) tetrathiafulvalene undoped molecule (a) such as the bond lengths and bond angles by B3LYP, RHF and wB97XD methods with the same basis set cc-pVDZ are given in Table 1; the experimental results [23] are also in this Table 1 for comparison. Based on our calculations, the results obtained using B3LYP and wB97XD functionals are full agreement with experimental data [23] than the HF method. From this table, some of the calculated bond lengths for BEDT-TTF such as C1–C2, C3–C6 and C4–C5 (double bond length of carbon atom) with both DFT methods are in good agreement with experimental values reported by Guionneau et al [23]. Moreover, the largest difference between experimental and computed bond length is about 0.056 Å for B3LYP, 0.037 Å for wB97XD and 0.042 Å for RHF. On the other hand, the smallest difference is about 0.001 Å for B3LYP, 0.0003 Å for wB97XD and -0.039 Å for RHF. These bond lengths show a slow variation from the uncorrelated level of theory (RHF) to the correlated level (B3LYP and wB97XD). In fact, it is well known that the Hartree-Fock method under-estimates bond lengths and the inclusion of the electron correlation make them longer [24]. This elongation usually makes a better agreement between theory and experiment. The same observation has been done in this work. Most of the bond lengths and angles obtained with B3LYP and wB97XD are very similar to the values reported by Guionneau et al [23], by Imamura et al [3] and by Demiralp et al [4]. However, we observe the large difference between C18–S8 bond length values obtained with the three methods.

The bond angles (^o^) of the undoped molecule follow the same pattern. The calculated bond angles were slightly equal to the values listed by Guionneau et al [23]. We can conclude that the experimental results and the theoretical calculations have a better agreement.

### 3.2 Natural bond orbital (NBO) analysis

NBO analysis has been performed on the both doped structures ($C_{7}H_{5}B_{3}S_{8}$ and $C_{8}H_{6}B_{2}S_{8}$) in order to explain the intermolecular and intramolecular bonding, transfer of charge, stability and conjugated interactions [25]. The second order perturbation theory of Fock matrix was carried out to estimate the donor-acceptor interactions in NBO studies [26]. For each donor (i) and acceptor (j), the stabilization energy E(2) linked with the delocalization value of i,j is evaluated by these mathematical formulae (see Eq. (1)) [25]:

(1) $$E\left(2\right)=\Delta E_{ij}=q_{i}\frac{F{\left(i,j\right)}^{2}\;}{E_{i}-E_{j}}$$

Where: $q_{i}$ represent the donor orbital occupancy status

$E_{i}$ and $E_{j}$ represent the donor and acceptor orbital energy values respectively;

F(i,j) is Fock matrix elements.

In the present work, the NBO calculations were investigated using B3LYP/cc-pVDZ level of theory and summarized in Tables 2(a) and (b) for both the doped systems. This analysis is carried out in order to examine all interactions between donor (filled) Lewis-type NBOs and acceptor (empty) non-Lewis NBOs and estimate their energetic importance.

**Table 2 (a):** Second-order perturbation theory of Fock matrix in NBO basis for $C_{7}H_{5}B_{3}S_{8}$.

Donor (i)	type	occupancy	Acceptor (j)	type	Occupancy	E(2)^a^ kcal/mol	E_i_-E_j_^b^ a.u	F(i,j)^c^ a.u
C1–C2	σ	1.99034	C1–S8	σ∗	0.04321	1.37	1.00	0.033
C1–C2	σ	1.99034	C1–S12	σ∗	0.03859	0.64	0.98	0.023
C1–C2	σ	1.99034	C2–S7	σ∗	0.04322	1.37	1.00	0.033
C1–C2	σ	1.99034	C2–S11	σ∗	0.03820	0.65	0.98	0.023
C1–C2	π	1.91975	S7–B18	π∗	0.01181	1.85	0.32	0.022
C1–C2	π	1.91975	S8–B20	π∗	0.01181	1.85	0.32	0.022
C1–S8	σ	1.97659	C1–C2	σ∗	0.03940	3.42	1.27	0.059
C1–S8	σ	1.97659	C2–S11	σ∗	0.03820	3.80	0.86	0.051
C1–S8	σ	1.97659	C 3–S12	σ∗	0.04364	0.61	0.86	0.021
C1–S8	σ	1.97659	B20–H21	σ∗	0.00833	0.88	1.19	0.029
C1–S12	σ	1.96758	C1–C2	σ∗	0.03859	2.00	1.24	0.045
C1–S12	σ	1.96758	C2–S7	σ∗	0.04322	6.15	0.85	0.065
C1–S12	σ	1.96758	C3–C6	σ∗	0.04490	2.28	1.26	0.048
C1–S12	σ	1.96758	C3–C6	π∗	0.37743	0.84	0.67	0.023
C1–S12	σ	1.96758	S 8–B20	σ∗	0.01181	1.32	0.94	0.032
C2–S7	σ	1.976559	C1–C2	σ∗	0.03940	3.42	1.27	0.059
C2–S 7	σ	1.976559	C1–S12	σ∗	0.03859	3.80	0.86	0.051
C2–S7	σ	1.976559	C 3–S11	σ∗	0.04384	0.61	0.86	0.021
C2–S7	σ	1.976559	B18–H19	σ∗	0.00833	0.88	1.19	0.029
C2–S11	σ	1.96760	C1–C2	σ∗	0.03859	2.00	1.24	0.045
C2–S11	σ	1.96760	C1–S 8	σ∗	0.04321	6.15	0.85	0.065
C2–S11	σ	1.96760	C 3–C 6	σ∗	0.04490	2.28	1.26	0.048
C 2–S11	σ	1.96760	C3–C6	π∗	0.37743	0.82	0.67	0.023
C2–S11	σ	1.96760	S7–B18	σ∗	0.07677	1.32	0.94	0.032
C3–C 6	σ	1.96257	C3–S11	σ∗	0.04384	0.97	0.97	0.028
C 3–C6	σ	1.99005	C3–S12	σ∗	0.04364	0.98	0.97	0.028
C3–C6	σ	1.99005	C 6–S13	σ∗	0.04691	0.92	0.97	0.027
C3–C6	σ	1.99005	C6–S14	σ∗	0.04310	0.92	0.97	0.027
C3–C 6	σ	1.99005	C5–S14	σ∗	0.04071	0.56	0.51	0.015
C3–S11	σ	1.97010	C2–S 7	σ∗	0.04322	2.79	0.85	0.044
C3–S11	σ	1.97010	C 3–C 6	σ∗	0.04490	2.16	1.27	0.047
C3–S 11	σ	1.97010	C6–S14	σ∗	0.04310	5.52	0.84	0.061
C3–S 12	σ	1.97018	C1–S8	σ∗	0.04321	2.79	0.85	0.044
C3–S12	σ	1.97018	C 3–C 6	σ∗	0.04490	2.17	1.27	0.047
C3–S 12	σ	1.97018	C6–S 13	σ∗	0.04691	5.51	0.84	0.061
C4–C5	σ	1.99008	C4–S9	σ∗	0.03222	1.31	0.99	0.032
C4–C5	σ	1.99008	C4–S13	σ∗	0.04451	0.72	0.98	0.024
C 4–C5	σ	1.99008	C 5–S 10	σ∗	0.03797	1.22	0.98	0.031
C4–C5	σ	1.99008	C5–S14	σ∗	0.04071	0.74	0.98	0.024
C 4–C5	π	1.96426	S 10–B 22	π∗	0.06517	1.37	0.31	0.019
C 4–S9	σ	1.97446	C 4–C 5	σ∗	0.04081	3.24	1.27	0.058
C 4–S9	σ	1.97446	C 5–S 14	σ∗	0.04071	4.26	0.85	0.054
C 4–S9	σ	1.97446	C 6–S 13	σ∗	0.04691	0.63	0.84	0.021
C 4–S9	σ	1.97446	C 15–H 16	σ∗	0.01198	1.08	1.11	0.031
C 4–S 13	σ	1.96734	C 3–C 6	σ∗	0.04490	2.25	1.26	0.048
C 4–S 13	σ	1.96734	C 4–C 5	σ∗	0.04081	2.08	1.25	0.046
C 4–S 13	σ	1.96734	C 5–S 10	σ∗	0.03797	6.03	0.84	0.063
C 4–S 13	σ	1.96734	S 9–C 15	σ∗	0/01775	0.67	0.80	0.021
C 5–S 10	σ	1.97419	C 4–C 5	σ∗	0.04081	3.01	1.27	0.055
C 5–S 10	σ	1.97419	C 4–S 13	σ∗	0.04451	4.23	0.85	0.054
C 5–S 10	σ	1.97419	C 6–S 14	σ∗	0.04310	0.57	0.84	0.020
C 5–S 10	σ	1.97419	B 22–H 23	σ∗	0.00921	1.56	1.18	0.038
C 5–S 14	σ	1.96874	C 3–C 6	σ∗	0.04490	2.27	1.26	0.048
C 5–S 14	σ	1.96874	C 4–C 5	σ∗	0.04081	2.03	1.25	0.045
C 5–S 14	σ	1.96874	C 4–S 9	σ∗	0.03222	5.84	0.85	0.063
C 5–S 14	σ	1.96874	S 10–B 22	σ∗	0.01511	1.09	0.92	0.028
C 6–S 13	σ	1.96925	C 3–C 6	σ∗	0.04490	2.12	1.26	0.046
C 6–S 13	σ	1.96925	C 3–S 12	σ∗	0.03859	5.66	0.83	0.061
C 6–S 13	σ	1.96925	C 4–S 9	σ∗	0.03222	3.08	0.85	0.046
C 6–S 14	σ	1.96919	C 3–C 6	σ∗	0.04490	2.13	1.26	0.046
C 6–S 14	σ	1.96919	C 3–S 11	σ∗	0.04384	5.66	0.83	0.061
C 6–S 14	σ	1.96919	C 5–S 10	σ∗	0.03797	3.13	0.84	0.046
S 7–B 18	σ	1.98201	C 2–S 11	σ∗	0.03820	3.04	0.83	0.045
S 7–B 18	σ	1.98201	B 20–H 21	σ∗	0.00833	1.59	1.17	0.039
S 7–B 18	π	1.87061	C 1–C 2	π∗	0.36401	15.54	0.27	0.061
S 7–B 18	π	1.87061	S 7–B 18	π∗	0.01181	0.60	0.29	0.012
S 7–B 18	π	1.87061	S 8–B 20	π∗	0.07676	4.41	0.29	0.032
S 8–B 20	σ	1.98202	C 1–S 12	σ∗	0.03859	3.04	0.83	0.045
S 8–B 20	σ	1.98202	B 18–H 19	σ∗	0.00833	1.59	1.17	0.039
S 8–B 20	π	1.87055	C 1–C 2	π∗	0.36401	15.55	0.27	0.061
S 8–B 20	π	1.87055	S 7–B 18	π∗	0.01181	4.41	0.29	0.032
S 8–B 20	π	1.87055	S 8–B 20	π∗	0.07676	0.60	0.29	0.012
S 9–C 15	σ	1.97060	C 4–S 13	σ∗	0.04451	2.41	0.76	0.038
S 9–C 15	σ	1.97060	C 15–B 22	σ∗	0.01724	0.70	0.97	0.023
S 9–C 15	σ	1.97060	B 22–H 23	σ∗	0.00921	1.77	1.09	0.039
S 10–B 22	σ	1.97724	C 5–S 14	σ∗	0.04071	3.54	0.82	0.048
S 10–B 22	σ	1.97724	C 15–H 16	σ∗	0.01198	1.55	1.08	0.037
S 10–B22	π	1.92519	C 4–C 5	π∗	0.35776	12.67	0.28	0.057
C 15–H 16	σ	1.97563	C 4–S 9	σ∗	0.03222	1.73	0.71	0.031
C 15–H16	σ	1.97563	S 10–B 22	σ∗	0.01511	2.66	0.78	0.041
C15–H 16	σ	1.97563	C 15–B 22	σ∗	0.06517	0.95	0.90	0.026
C15–H17	σ	1.97498	C 15–B 22	σ∗	0.06517	0.79	0.90	0.024
C15–H 17	σ	1.97498	B 22–H 23	σ∗	0.00921	0.64	1.02	0.023
C15–B 22	σ	1.98864	C 15–H 16	σ∗	0.01198	1.37	1.01	0.033
C15–B 22	σ	1.98864	C 15–H 17	σ∗	0.01337	1.52	1.00	0.035
B18–H 19	σ	1.96952	C 2–S 7	σ∗	0.04322	3.96	0.60	0.044
B18–H 19	σ	1.96952	S 8–B 20	σ∗	0.07676	2.78	0.69	0.039
B20–H 21	σ	1.96952	C 1–S 8	σ∗	0.04321	3.96	0.60	0.044
B20–H 21	σ	1.96952	S 7–B 18	σ∗	0.07677	2.78	0.69	0.039
B 22–H 23	σ	1.97342	C 5–S 10	σ∗	0.03797	2.89	0.60	0.037
B22–H 23	σ	1.97342	S 9–C 15	σ∗	0.01775	2.28	0.57	0.032
B 22–H 23	σ	1.97342	C 15–H 17	σ∗	0.02153	0.70	0.85	0.022
S 8	LP (1)	1.96796	C 1–C 2	σ∗	0.03859	6.93	1.17	0.080
S 9	LP (1)	1.97694	C 4–C 5	σ∗	0.04081	4.80	1.24	0.069
S 9	LP (2)	1.83903	C 4–C 5	σ∗	0.04081	1.36	0.84	0.031
S 9	LP (2)	1.83903	C 4–C 5	π∗	0.35776	16.17	0.23	0.058
S 9	LP (2)	1.83903	C 4–S 13	σ∗	0.04451	2.79	0.42	0.032
S 10	LP (1)	1.96598	C 4–C 5	σ∗	0.04081	5.44	1.19	0.072
S 11	LP (2)	1.78106	C 1–C 2	π∗	0.36401	20.64	0.23	0.064
S 11	LP (2)	1.78106	C 3–C 6	π∗	0.37743	14.96	0.26	0.058
S 12	LP (2)	1.78077	C 1–C 2	π∗	0.36401	20.66	0.23	0.064
S 12	LP (2)	1.78077	C 3–C 6	π∗	0.37743	15.01	0.26	0.058
S 13	LP (1)	1.97254	C 4–C 5	σ∗	0.04081	3.06	1.25	0.055
S 13	LP (2)	1.77831	C 3–C 6	π∗	0.37743	14.28	0.26	0.056
S 13	LP (2)	1.77831	C4–C5	π∗	0.35776	20.41	0.24	0.064
S 14	LP (1)	1.97248	C 4–C 5	σ∗	0.04081	2.98	1.25	0.055
S 14	LP (1)	1.97248	C 6–S 13	σ∗	0.04691	3.36	0.83	0.047
S14	LP (2)	1.78832	C3–C6	σ∗	0.04490	1.36	0.85	0.032
S14	LP (2)	1.78832	C3–C6	π∗	0.37743	14.32	0.26	0.056
S14	LP (2)	1.78832	C4–C5	π∗	0.35776	18.92	0.23	0.062
S14	LP (2)	1.78832	C6–S13	σ∗	0.04691	1.09	0.42	0.020

a E(2) means energy of hyper conjugative interaction (stabilization energy).

b Energy difference between donor and acceptor i and j NBO orbitals.

c F(i,j) is the Fock matrix element between i and j NBO orbitals.

**Table 2 (b):** Second-order perturbation theory of Fock matrix in NBO basis for $C_{8}H_{6}B_{2}S_{8}$.

Donor (i)	type	occupancy	Acceptor (j)	type	Occupancy	E(2)^a^ kcal/mol	E_i_-E_j_^b^ a.u	F(i,j)^c^ a.u
C 1–C 2	σ	1.99035	C 1–S 8	σ∗	0.04326	1.37	1.00	0.033
C 1–C 2	σ	1.99035	C 1–S 12	σ∗	0.03860	0.65	0.98	0.023
C 1–C 2	σ	1.99035	C 2–S 7	σ∗	0.04323	1.37	1.00	0.033
C 1–C 2	σ	1.99035	C 2–S 11	σ∗	0.03822	0.65	0.98	0.023
C 1–C 2	π	1.91921	S 7–B 18	π∗	0.07744	1.86	0.32	0.022
C 1–C 2	π	1.91921	S 8–B 20	π∗	0.07744	1.86	0.32	0.022
C 1–S 8	σ	1.97668	C 1–C 2	σ∗	0.03939	3.41	1.27	0.059
C 1–S 8	σ	1.97668	C 2–S 11	σ∗	0.03822	3.79	0.86	0.051
C 1–S 8	σ	1.97668	C 3–S 12	σ∗	0.04381	0.61	0.86	0.021
C 1–S 8	σ	1.97668	B 20–H 21	σ∗	0.00834	0.88	1.19	0.029
C 1–S 12	σ	1.96758	C 1–C 2	σ∗	0.03939	2.01	1.24	0.045
C 1–S 12	σ	1.96758	C 2–S 7	σ∗	0.04323	6.16	0.85	0.065
C 1–S 12	σ	1.96758	C 3–C 6	σ∗	0.04510	2.27	1.26	0.048
C 1–S 12	σ	1.96758	C 3–C 6	π∗	0.37850	0.84	0.67	0.023
C 1–S 12	σ	1.96758	S 8–B 20	σ∗	0.01183	1.32	0.94	0.032
C 2–S 7	σ	1.97664	C 1–C 2	σ∗	0.03939	3.41	1.27	0.059
C 2–S 7	σ	1.97664	C 1–S 12	σ∗	0.03860	3.79	0.86	0.051
C 2–S 7	σ	1.97664	C 3–S 11	σ∗	0.04367	0.61	0.86	0.021
C 2–S 7	σ	1.97664	B 18–H 19	σ∗	0.00834	0.88	1.19	0.029
C 2–S 11	σ	1.96756	C 1–C 2	σ∗	0.03939	2.01	1.24	0.045
C 2–S 11	σ	1.96756	C 1–S 8	σ∗	0.04326	6.17	0.85	0.065
C 2–S 11	σ	1.96756	C 3–C 6	σ∗	0.04510	2.27	1.26	0.048
C 2–S 11	σ	1.96756	C 3–C 6	π∗	0.37850	0.83	0.67	0.023
C 2–S 11	σ	1.96756	S 7–B 18	σ∗	0.01182	1.32	0.94	0.032
C 3–C 6	σ	1.99004	C 3–S 11	σ∗	0.04367	0.97	0.97	0.028
C 3–C 6	σ	1.99004	C 3–S 12	σ∗	0.04381	0.98	0.97	0.028
C 3–C 6	σ	1.99004	C 6–S 13	σ∗	0.04607	0.92	0.97	0.027
C 3–C 6	σ	1.99004	C 6–S 14	σ∗	0.04433	0.92	0.97	0.027
C 3–C 6	π	1.96263	C 1–S 12	σ∗	0.03860	0.51	0.50	0.014
C 3–C 6	π	1.96263	C 2–S 11	σ∗	0.03822	0.52	0.50	0.014
C 3–C 6	π	1.96263	C 4–S 13	σ∗	0.04649	0.53	0.51	0.015
C 3–C 6	π	1.96263	C 5–S 14	σ∗	0.03888	0.58	0.51	0.015
C 3–S 12	σ	1.97028	C 1–S 8	σ∗	0.04326	2.80	0.85	0.044
C 3–S 12	σ	1.97028	C 3–C 6	σ∗	0.04510	2.15	1.27	0.047
C 3–S 12	σ	1.97028	C 6–S 13	σ∗	0.04607	5.47	0.84	0.061
C 4–C 5	σ	1.99019	C 4–S 9	σ∗	0.03132	1.35	1.00	0.033
C 4–C 5	σ	1.99019	C 4–S 13	σ∗	0.04649	0.74	0.98	0.024
C 4–C 5	σ	1.99019	C 5––S 10	σ∗	0.03441	1.17	0.99	0.030
C 4–C 5	σ	1.99019	C 5–S 14	σ∗	0.03888	0.74	0.98	0.024
C 4–C 5	π	1.96745	S 9–C 15	σ∗	0.02987	0.95	0.45	0.018
C 4–S 9	σ	1.97374	C 4–C 5	σ∗	0.03994	3.29	1.27	0.058
C 4–S 9	σ	1.97374	C 5–S 14	σ∗	0.03888	4.37	0.85	0.054
C 4–S 9	σ	1.97374	C 6–S 13	σ∗	0.04607	0.63	0.84	0.021
C 4–S 9	σ	1.97374	C 15–H 16	σ∗	0.00924	1.15	1.10	0.032
C 4–S 13	σ	1.96798	C 3–C 6	σ∗	0.04510	2.28	1.26	0.048
C 4–S 13	σ	1.96798	C 3–C 6	π∗	0.37850	0.92	0.66	0.024
C 4–S 13	σ	1.96798	C 4–C 5	σ∗	0.03994	2.08	1.25	0.046
C 4–S 13	σ	1.96798	C 5–S 10	σ∗	0.03441	5.97	0.84	0.063
C 4–S 13	σ	1.96798	S 9–C 15	σ∗	0.02987	0.67	0.79	0.021
C 5–S 10	σ	1.97317	C 4–C 5	σ∗	0.03994	3.06	1.27	0.056
C 5–S 10	σ	1.97317	C 4–S 13	σ∗	0.04649	4.42	0.85	0.055
C 5–S 10	σ	1.97317	C 6–S 14	σ∗	0.04433	0.60	0.84	0.020
C 5–S 10	σ	1.97317	S 10–C 22	σ∗	0.02157	0.62	0.85	0.020
C 5–S 10	σ	1.97317	C 22–H 23	σ∗	0.01928	0.72	1.14	0.026
C 5–S 10	σ	1.97317	C 22–H 24	σ∗	0.01944	0.75	1.14	0.026
C 5–S 14	σ	1.96806	C 3–C 6	σ∗	0.04510	2.25	1.26	0.048
C 5–S 14	σ	1.96806	C 3–C 6	π∗	0.37850	0.93	0.66	0.024
C 5–S 14	σ	1.96806	C 4–C 5	σ∗	0.03994	2.04	1.26	0.045
C 5–S 14	σ	1.96806	C 4–S 9	σ∗	0.03132	5.83	0.85	0.063
C 5–S 14	σ	1.96806	S 10–C 22	σ∗	0.02157	0.98	0.83	0.026
C 6–S 13	σ	1.96893	C 3–C 6	σ∗	0.04510	2.14	1.26	0.046
C 6–S 13	σ	1.96893	C 3–S 12	σ∗	0.04381	5.71	0.83	0.062
C 6–S 13	σ	1.96893	C 4–S 9	σ∗	0.03132	3.11	0.85	0.046
C 6–S 14	σ	1.96896	C 3–C 6	σ∗	0.04510	2.10	1.26	0.046
C 6–S 14	σ	1.96896	C 3–S 11	σ∗	0.04367	5.66	0.83	0.061
C 6–S 14	σ	1.96896	C 5–S 10	σ∗	0.03441	3.12	0.84	0.046
S 7–B 18	σ	1.98208	C 2–S 11	σ∗	0.03822	3.03	0.83	0.045
S 7–B 18	σ	1.98208	B 20–H 21	σ∗	0.00834	1.59	1.17	0.039
S 7–B 18	π	1.87086	C 1–C 2	π∗	0.36429	15.50	0.27	0.061
S 7–B 18	π	1.87086	S 7–B 18	π∗	0.07744	0.61	0.29	0.012
S 7–B 18	π	1.87086	S 8–B 20	π∗	0.07744	4.42	0.29	0.032
S 8–B 20	σ	1.98208	C 1–S 12	σ∗	0.03860	3.03	0.83	0.045
S 8–B 20	σ	1.98208	B 18–H 19	σ∗	0.00834	1.59	1.17	0.039
S 8–B 20	π	1.87085	C 1–C 2	π∗	0.36429	15.50	0.27	0.061
S 8–B 20	π	1.87085	S 7–B 18	π∗	0.07744	4.42	0.29	0.032
S 8–B 20	π	1.87085	S 8–B 20	π∗	0.07744	0.61	0.29	0.012
S 9–C 15	σ	1.97492	C 4–C 5	π∗	0.37201	1.43	0.58	0.028
S 9–C 15	σ	1.97492	C 4–S 13	σ∗	0.04649	2.27	0.77	0.038
S 9–C 15	σ	1.97492	C 22–H 23	σ∗	0.01928	1.81	1.07	0.039
C 15–H 16	σ	1.97682	C 4–S 9	σ∗	0.03132	1.61	0.73	0.031
C 15–H 16	σ	1.97682	S 10–C 22	σ∗	0.02157	4.31	0.71	0.049
C 15–H 17	σ	1.99014	C 22–H 24	σ∗	0.01944	1.39	1.00	0.033
B 18–H 19	σ	1.96961	C 2–S 7	σ∗	0.04323	3.95	0.60	0.044
B 18–H 19	σ	1.96961	S 8–B 20	σ∗	0.01183	2.78	0.69	0.039
B 20–H 21	σ	1.96962	C 1–S 8	σ∗	0.04326	3.95	0.60	0.044
B 20–H 21	σ	1.96962	S 7–B 18	σ∗	0.01182	2.78	0.69	0.039
C 22–H 23	σ	1.97504	C 5–S 10	σ∗	0.03441	1.02	0.72	0.024
C 22–H 23	σ	1.97504	S 9–C 15	σ∗	0.02987	4.32	0.67	0.048
C 22–H 24	σ	1.98241	C 5–S 10	σ∗	0.03441	0.68	0.71	0.020
C 22–H 24	σ	1.98241	C 15–H 17	σ∗	0.02207	3.59	0.96	0.052
S 7	LP (1)	1.96802	C 1–C 2	σ∗	0.03939	6.92	1.17	0.080
S 7	LP (1)	1.96802	B 18–H 19	σ∗	0.00834	0.57	1.09	0.022
S 7	LP (1)	1.96802	B 18–B 20	σ∗	0.01339	3.01	1.04	0.050
S 8	LP (1)	1.96802	C 1–C 2	σ∗	0.03939	6.92	1.17	0.080
S 8	LP (1)	1.96802	B 18–B 20	σ∗	0.01339	3.01	1.04	0.050
S 8	LP (1)	1.96802	B 20–H 21	σ∗	0.00834	0.57	1.09	0.022
S 9	LP (1)	1.97732	C 4–C 5	σ∗	0.03994	4.74	1.24	0.069
S 9	LP (1)	1.97732	C 15–H 17	σ∗	0.02207	0.62	1.07	0.023
S 9	LP (1)	1.97732	C 15–C 22	σ∗	0.02966	1.19	0.94	0.030
S 9	LP (2)	1.97732	C 4–C 5	σ∗	0.03994	1.73	0.85	0.035
S 9	LP (2)	1.97732	C 4–C 5	π∗	0.37201	12.98	0.23	0.052
S 9	LP (2)	1.97732	C 4–S 13	σ∗	0.04649	3.25	0.43	0.034
S 9	LP (2)	1.97732	C 6–S 13	σ∗	0.04607	0.52	0.42	0.013
S 9	LP (2)	1.97732	C 15–H 17	σ∗	0.02207	4.03	0.68	0.048
S 9	LP (2)	1.97732	C 15–C 22	σ∗	0.02966	3.16	0.55	0.038
S 9	LP (2)	1.97732	C 22–H 23	σ∗	0.01928	0.82	0.72	0.022
S 10	LP (1)	1.97275	C 4–C 5	σ∗	0.03994	5.00	1.22	0.070
S 10	LP (1)	1.97275	C 15–C 22	σ∗	0.02966	4.23	0.92	0.056
S 10	LP (2)	1.97275	C 4–C 5	σ∗	0.03994	0.58	0.85	0.020
S 10	LP (2)	1.97275	C 4–C 5	π∗	0.37201	18.38	0.24	0.063
S 10	LP (2)	1.97275	C 5–S 14	σ∗	0.03888	1.21	0.42	0.021
S 10	LP (2)	1.97275	C 22–H 23	σ∗	0.01928	3.22	0.72	0.045
S 10	LP (2)	1.97275	C 22–H 24	σ∗	0.01944	4.60	0.72	0.053
S 11	LP (1)	1.97208	C 1–C 2	σ∗	0.03939	3.10	1.24	0.056
S 11	LP (1)	1.97208	C 3–S 12	σ∗	0.04381	3.47	0.83	0.048
S 11	LP (2)	1.78173	C 1–C 2	π∗	0.36429	20.69	0.23	0.064
S 11	LP (2)	1.78173	C 3–C 6	σ∗	0.04510	1.20	0.85	0.030
S 11	LP (2)	1.78173	C 3–C 6	π∗	0.37850	14.76	0.26	0.058
S 11	LP (2)	1.78173	C 3–S 12	σ∗	0.04381	0.82	0.42	0.017
S 12	LP (1)	1.97207	C 1–C 2	σ∗	0.03939	3.10	1.24	0.056
S 12	LP (1)	1.97207	C 3–S 11	σ∗	0.04367	3.48	0.83	0.048
S 12	LP (2)	1.78199	C 1–C 2	π∗	0.36429	20.68	0.23	0.064
S 12	LP (2)	1.78199	C 3–C 6	σ∗	0.04510	1.19	0.85	0.030
S 12	LP (2)	1.78199	C 3–C 6	π∗	0.37850	14.79	0.26	0.058
S 12	LP (2)	1.78199	C 3–S 11	σ∗	0.04367	0.81	0.42	0.017
S 13	LP (1)	1.97220	C 4–C 5	σ∗	0.03994	3.04	1.25	0.055
S 13	LP (1)	1.97220	C 6–S 14	σ∗	0.04433	3.39	0.83	0.047
S 13	LP (2)	1.78677	C 3–C 6	σ∗	0.04510	1.32	0.85	0.031
S 13	LP (2)	1.78677	C 3–C 6	π∗	0.37850	14.53	0.25	0.057
S 13	LP (2)	1.78677	C 4–C 5	π∗	0.37201	19.94	0.23	0.064
S 13	LP (2)	1.78677	C 6–S 14	σ∗	0.04433	1.06	0.42	0.020
S 14	LP (1)	1.97266	C 4–C 5	σ∗	0.03994	3.02	1.26	0.055
S 14	LP (1)	1.97266	C 6–S 13	σ∗	0.04607	3.34	0.83	0.047
S 14	LP (2)	1.78820	C 3–C 6	σ∗	0.04510	1.39	0.85	0.032
S 14	LP (2)	1.78820	C 3–C 6	π∗	0.37850	14.23	0.26	0.056
S 14	LP (2)	1.78820	C 4–C 5	π∗	0.37201	19.44	0.24	0.063
S 14	LP (2)	1.78820	C 6–S 13	σ∗	0.04607	1.08	0.42	0.020

a E(2) means energy of hyper conjugative interaction (stabilization energy).

b Energy difference between donor and acceptor i and j NBO orbitals.

c F(i,j) is the Fock matrix element between i and j NBO orbitals.

In Table 2(a) S8–B20 shows the highest E(2) value of 15.55 kcal/mol in π to π∗ transition. C1–S12 and C2–S11 show the highest E(2) values of 6.15 kcal/mol in σ to σ∗ transition. We also obtained a stabilization energy of 15.54 kcal/mol for S7–B18 (π) to C1–C2 (π∗) transition and 12.67 kcal/mol for S10–B12 (π) to C4–C5 (π∗) transition. Another π to π∗ transition is observed at C1–C2 to S7–B18 with 1.85 kcal/mol. For transition C4–S13 (σ) to C5–S10 (σ∗); C6–S13 (σ) to C3–S12 (σ∗) and C3–S11 (σ) to C6–S14 (σ∗) stabilizations energies are 6.03 kcal/mol; 5.66 kcal/mol and 5.52 kcal/mol respectively. Noted that the lone pairs in the sulfur atoms participate in the stabilization of the molecule by LP(2) to π∗ and LP(2) to σ∗ interactions with considerable stabilization like the highest E(2) value of 20.66 kcal/mol in LP(2) to π∗ transition.

The calculated values E(2) of the second proposed material B2-doped molecule are shown in Table 2(b). S8–B20 and S7–B18 show the highest E(2) value of 15.50 kcal/mol in π to π∗ transition. C2–S11 shows the highest E(2) value of 6.17 kcal/mol inσ to σ∗ transition. A stabilization energy of 4.42 kcal/mol is observed for S8–B20 (π) to S7–B18 (π∗) and 1.86 kcal/mol for C1–C2 (π) to S8–B20 (π∗) transition. For transitions C1–S12 (σ) to C2–S7 (σ∗); C4–S13 (σ) to C5–S10 (σ∗) and C3–S12 (σ) to C6–S13 (σ∗) stabilizations energies are 6.17 kcal/mol, 5.59 kcal/mol and 5.47 kcal/mol respectively. LP(2)→σ∗ and LP(2)→π∗ interactions also participate in the stabilization of the system. The highest E(2) value of 20.69 kcal/mol is shown in LP(2) to π∗ transition.

The π→π∗ transitions in both the doped molecules account for the high polarization and which is further responsible for the NLO activity of the new molecules.

### 3.3 Nonlinear optical properties of the molecules

The non-linear optical parameters are useful in current technologies such as communication and telecommunication [25, 27, 28]. Nonlinear optical properties are very important for the molecular structure and organic materials [29, 30, 31, 32]. The Parameters, such as first hyperpolarizability ($\beta_{mol}$), polarizability (<α>) and the dipole moment (μ) of BEDT-TTF and their derivatives were evaluated using RHF, B3LYP and wB97XD methods with the cc-pVDZ. The anisotropy of the polarizability (Δα) and some NLO properties can be calculated using the following Eqs. (2), (3), (4), and (5) [33, 34, 35, 36] and summarized in Table 3.

(2) $$\mu_{tot}=\sqrt{\left(\mu_{x}^{2}+\mu_{y}^{2}+\mu_{z}^{2}\right)}$$

(3) $$<\alpha >=\frac{1}{3}\left(\alpha_{xx}+\alpha_{yy}+\alpha_{zz}\right)$$

(4) $$\Delta \alpha =\frac{1}{\sqrt{2}}{\left[{\left(\alpha_{xx}-\alpha_{yy}\right)}^{2}+{\left(\alpha_{yy}-\alpha_{zz}\right)}^{2}+{\left(\alpha_{zz}-\alpha_{xx}\right)}^{2}+6\left(\alpha_{xz}^{2}+\alpha_{xy}^{2}+\alpha_{yz}^{2}\right)\right]}^{\frac{1}{2}}$$

(5) $$\beta_{mol}={\left[{\left(\beta_{xxx}+\beta_{xyy}+\beta_{xzz}\right)}^{2}+{\left(\beta_{xxy}+\beta_{yyy}+\beta_{yzz}\right)}^{2}+{\left(\beta_{zxx}+\beta_{zyy}+\beta_{zzz}\right)}^{2}\right]}^{\frac{1}{2}}$$

**Table 3:** Calculated values of dipole moment μ (Debye), average polarizability <α> × 10^−24^(e.s.u), anisotropy $\Delta \alpha \times 10^{-24}$ (esu)and first order hyperpolarizability $\beta_{mol}\left(\times 10^{-33}\text{esu}\right)$ of **C**_**10**_**H**_**8**_**S**_**8**_**, C**_**7**_**B**_**3**_**H**_**8**_**S**_**8**_ and **C**_**8**_**B**_**2**_**H**_**8**_**S**_**8**_ obtained employing RHF, B3LYP and Wb97XD with cc-pVDZ basis set.

Molecules parameters	$C_{10}H_{8}S_{8}$			$C_{7}B_{3}H_{5}S_{8}$			$C_{8}B_{2}H_{6}S_{8}$		
	B3LYP	wB97XD	RHF	B3LYP	wB97XD	RHF	B3LYP	wB97XD	RHF
**μ (Debye)**	**1.41**	**1.55**	**0.97**	**2.25**	**1.94**	**1.30**	**3.82**	**3.33**	**2.48**
$\alpha_{xx}$	231.32	413.60	223.01	482.55	446.03	404.48	474.67	438.62	395.17
$\alpha_{xy}$	2.80	-1.43	1.33	1.06	-0.48	-0.82	-0.20	-17	-0.10
$\alpha_{yy}$	393.01	226.12	335.46	239.68	234.20	225.10	238.64	233.33	226.49
$\alpha_{xz}$	-1.09	-0.004	-0.11	-14.09	12.73	10.28	16.82	14.83	8.67
$\alpha_{yz}$	-113.66	-0.005	-93.01	-0.58	-0.71	-0.63	-1.42	-1.47	-1.94
$\alpha_{zz}$	172.17	122.22	156.21	120.68	119.21	112.87	121.51	120.10	114.41
$<\alpha >10^{-24}$**(esu)**	**39.35**	**37.67**	**35.305**	**41.64**	**39.49**	**36.68**	**41.24**	**39.13**	**36.36**
**Δα**$*10^{-24}$**(esu)**	**41.387**	**26.81**	**33.344**	**33.57**	**30.18**	**26.76**	**32.79**	**29.43**	**25.70**
$\beta_{xxx}$	0.047	-0.0015	0.116	-2175.57	894.52	447.04	3182.32	1382.15	65.33
$\beta_{xxy}$	-9.337	-0.0007	-3.892	205.86	96.93	19.41	24.71	-1.43	1.03
$\beta_{xyy}$	-24.498	0.0004	-9.435	-94.75	84.83	45.63	180.55	152.85	96.74
$\beta_{yyy}$	-61.544	-0.0009	45.937	54.56	45.37	20.58	5.9	1.008	1.19
$\beta_{zxx}$	-22.04	-31.31	-9.385	770.87	388.22	197.48	651.22	319.15	202.33
$\beta_{xyz}$	-23.83	35.21	-8.903	37.61	-22.43	-7.78	19.56	17.38	6.14
$\beta_{zyy}$	-34.33	-18.75	26.344	52.86	45.75	27.79	21.61	19.57	11.18
$\beta_{yzz}$	24.47	-0.0007	79.041	-128.38	66.05	30.71	137.32	67.32	42.29
$\beta_{xzz}$	27.681	-0.0006	-16.52	3.65	1.44	-3.71	4.49	1.61	-2.09
$\beta_{zzz}$	-46.254	-28.94	42.98	41.98	22.74	3.44	53.78	39.63	50.64
$\beta \times 10^{-33}$**(esu)**	**973.45**	**682.51**	**716.07**	**22,149.35**	**9,933.81**	**4,944.48**	**30,885.46**	**14,223.54**	**7,215.52**

Noted that, α and β values of Gaussian output file are reported in atomic units (a.u), the calculated values were converted into electronic units (esu) using:

$\left(\text{α:}1.\text{a.u}=0.1482\times 10^{-24}\text{esuand }\beta :1.a.u=8.6393\times 10^{-33}\text{esu}\right).$

The μ, α and β of the doped molecules, computed using B3LYP/cc-pVDZ basis set, were found to be 2.25D, 41.64 × 10^−24^esu and 22,149.35 × 10^−33^esu respectively for C_7_B_3_H_5_S_8_and 3.82D, 41.24 × 10^−24^esuand 30,885.46 × 10^−33^esurespectivelyfor C_8_B_2_H_6_S_8_, while the obtained by using the wB97XD/cc-pVDZ are1.98D, 39.49 × 10^−24^esu and 9,933.81 × 10^−33^esu respectively for C_7_B_3_H_5_S_8_ and 3.33D, 39.13 × 10^−24^esuand 14,223.54 × 10^−33^esu respectively for C_8_B_2_H_6_S_8_. These values are given in Tables 2(a) and (b) and show that the molecules exhibits nonlinear behavior.

The dipole moment μ of the doped molecules (C_8_B_2_H_6_S_8_ and C_7_B_3_H_5_S_8_) are larger than those of the undoped BEDT-TTF molecule (C_10_H_8_S_8_) at all level of study. It is also observed that the $\beta_{mol}$ of C_7_B_3_H_5_S_8_ and C_8_B_2_H_6_S_8_ increase from the RHF level to the electron correlated method. These values are very large compared to those of undoped structure from the three methods. In this Table 3, we noticed that the values of the dipole moment are different from zero, which leads us to believe that these molecules are polar. On the other hand, those dipole moment values increase from the uncorrelated method to the correlated method for doped systems. Moreover, we observed an increase in the anisotropy of those undoped and doped systems in going from RHF to B3LYP and wB97XD.

On comparing the calculated first-order hyperpolarizability $\beta_{mol}$ values of doped molecules with boron with the corresponding value of Urea (*β*_*mol*_ = 928 × 10^−33^esu) [25]. The molecule of Urea is sometime used as reference molecule to study NLO properties of the compound [37]. The very high value of $\beta_{mol}$ = 22,149.35 × 10^−33^esu and $\beta_{mol}$ = 30,885.46 × 10^−33^esu using B3LYP/cc-pVDZ indicates that the two doped molecules (C_8_B_2_H_6_S_8_ and C_7_B_3_H_5_S_8_) can be a good NLO agent. Furthermore, the values of α and *β* of the molecules are greater than those reported in literature using other BEDT-TTF derivatives [13, 14].

### 3.4 Electronic properties

Energy gap is the difference between the lowest unoccupied molecular orbital (LUMO) and the highest occupied molecular orbital (HOMO) [25]. Electronic properties such as ionization potential (IP), electron affinity (AE), electronegativity (χ), global hardness (η), chemical softness (ϛ), chemical potential (μ) and Electrophilicity index (ω) were calculated and summarized in Table 4. The following equations (see Eq. (6)) are used to determine these parameters:

**Table 4:** Calculated Energy values of the molecules BEDT-TTF, 3B-, 2B- substituted C_10_H_8_S_8_ employing RHF, B3LYP and wB97XD methods by employing the cc-pVDZ basis set.

Molecules parameters	$C_{10}H_{8}S_{8}$			$C_{7}B_{3}H_{5}S_{8}$			$C_{8}B_{2}H_{6}S_{8}$		
	B3LYP	wB97XD	RHF	B3LYP	wB97XD	RHF	B3LYP	wB97XD	RHF
E_HOMO_ (eV)	-4.77	-6.68	-7.07	-5.20	-1.02	-7.34	-5.06	-6.94	-7.25
E_LUMO_ (eV)	-0.96	0.90	2.89	-2.81	-7.07	0.56	2.75	-0.96	0.59
Ionization potential	4.77	6.68	7.07	5.20	1.01	7.34	5.06	6.93	7.24
Electron affinity	0.96	-0.90	-2.89	2.81	7.07	-0.56	-2.75	0.96	-0.59
Energy gap (eV)	**3.81**	7.58	9.96	**2.39**	6.04	7.9	**2.31**	5.97	7.83
Electronegativity	2.86	2.89	2.09	4.00	4.04	3.39	1.16	3.95	3.33
Chemical potential	-2.86	-2.89	-2.09	-4.00	-4.04	-3.39	-1.16	-3.95	-3.33
Global hardness	1.91	3.79	4.98	1.20	3.02	3.95	1.16	2.98	3.92
Chemical Softness	0.52	0.26	0.20	0.84	0.33	0.25	0.87	0.34	0.26
Electrophilicity	2.14	1.10	0.44	6.70	2.69	1.46	0.58	2.61	1.41

The ionization potential (IP) and electron affinity (EA) can be expressed by HOMO and LUMO orbital energies as [37]:

(6) $$IP=-E_{HOMO}\;and\;EA=-E_{LUMO}$$

The electronegativity, based on the average of the electron affinity and ionization potential of molecules, energy gap, Softness, Electrophilicity index and the global hardness are given by the formulas [37, 38, 39, 40] (see Eqs. (7), (8), (9), (10), (11), and (12)):

(7) $$\text{Electronegativity}\left(\text{χ}\right)=-\frac{1}{2}\left(E_{LUMO}+E_{HOMO}\right)=-\text{μ}$$

(8) $$\text{Chemical potential}\left(\text{μ}\right)=\frac{1}{2}\left(E_{LUMO}+E_{HOMO}\right)$$

(9) $$\text{Global hardness}\left(\text{η}\right)=\frac{1}{2}\left(E_{LUMO}-E_{HOMO}\right)$$

(10) $$\text{Softness}\left(\text{ς}\right)=\frac{1}{\text{η}}\;$$

(11) $$\text{Energy gap}\left(E_{g}\right)=E_{LUMO}-E_{HOMO}$$

(12) $$\text{Electrophilicity index}\left(\text{ω}\right)=\frac{\mu^{2}}{2\text{η}}$$

Therefore, Egap is a very important parameter for the explaining stability and reactivity of molecule. A high ionization potential (IP) indicates high stability and hence chemical inertness, while a low ionization energy suggests a propensity of the molecule to reactivity. The electron affinity (AE) is defined as the energy released when an electron is added to a neutral molecule and hence a large (AE) value indicates the trend of the molecule to keep its electrons. A negative chemical potential (μ) indicates molecular stability or the difficulty of the molecule to decompose into its elements. The global electrophilicity index (ω) of a molecule is a measure of its stabilization energy following the addition of an external electronic charge or its resistance to exchange the electron with the system [36].

Based on our calculations, the energy gap changed from the RHF level to the B3LYP and wB97XD electron correlated methods. Our results show that when BEDT-TTF is substituted with boron atom. In this case, three atoms of carbon are replaced with three atoms of boron: C_7_ B_3_H_5_ S_8_ or with two atoms of carbon with two atoms of boron: C_8_B_2_H_6_ S_8_. HOMO-LUMO energy gap obtained using B3LYP level of theory with cc-pVDZ are respectively 2.39eV and 2.31eV less than 3eV, which makes them a good semiconductors materials. The Egap of semiconductor is less than 3eV [33], according to the band theory. We can conclude that the appropriate level of theory to study these molecules doped with boron is B3LYP because with RHF and wB97XD, the gap are rather of those of an insulator. We observed the global hardness, the ionization energy and chemical potential are bigger with RHF and smaller with B3LYP. The chemical softness and the electrophilicity index values are higher with the B3LYP and lower by using RHF. It is also mentioned that the high ionization potential (IP) computed with the three levels of theory and chemical potential (μ) indicate the stability and the reactivity, it means that, the undoped and doped molecules will not spontaneously decompose into its elements. Finally, due to the small values of energy gap of 3B- and 2B- doped BEDT-TTF we can conclude that these new materials are a good candidate for electronic applications in the fields of electronic devices.

#### 3.4.1 HOMO and LUMO diagram

**Figure 3:**
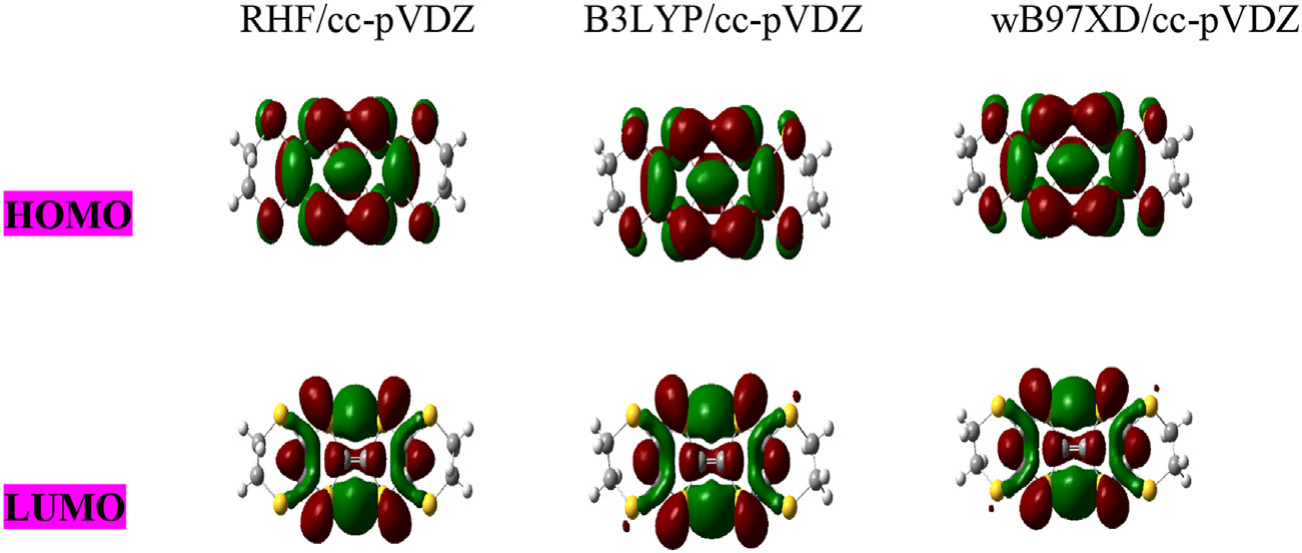
HOMO-LUMO diagram for C_10_H_8_S_8_ obtained at RHF, B3LYP and wB97XD methods using cc-pVDZ basis set.

**Figure 4:**
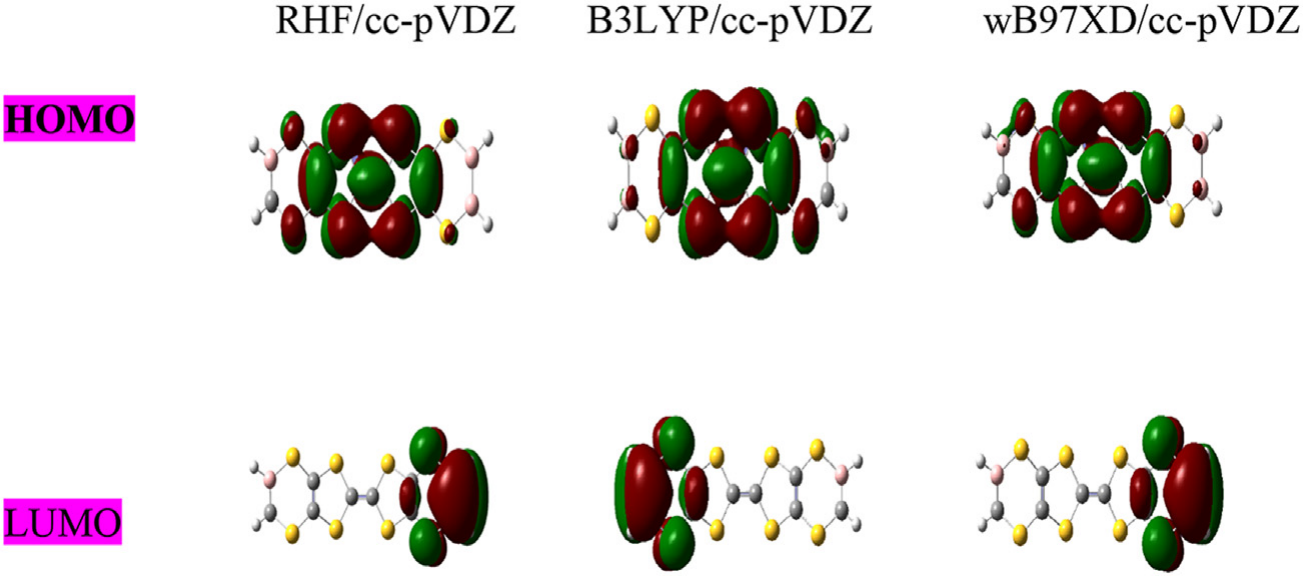
HOMO-LUMO diagram for C_7_ B_3_H_5_ S_8_ obtained at RHF, B3LYP and wB97XD methods using cc-pVDZ basis set.

**Figure 5:**
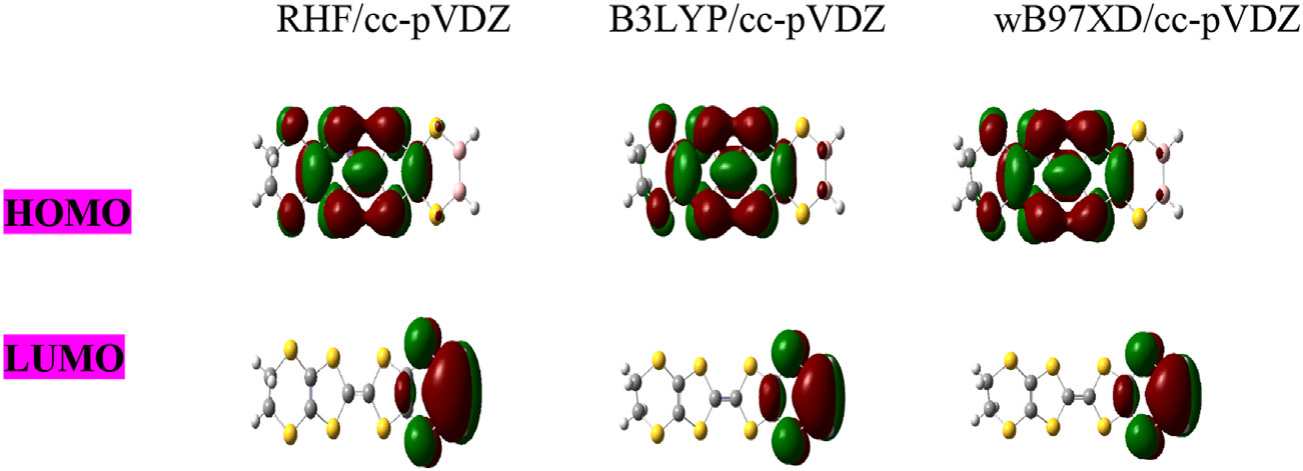
HOMO-LUMO diagram for C_8_B_2_H_6_S_8_.

The HOMO and LUMO molecular orbitals of C_10_H_8_S_8_, C_7_ B_3_H_5_ S_8_andC_8_B_2_H_6_ S_8_ are shown in Figures 3, 4, and 5 respectively. First, we observed from HOMO, LUMO diagrams of undoped molecule C_10_H_8_S_8_ obtained using the RHF, B3LYP, and wB97XD methods with cc-pVDZ are the same in Figure 3. On the other hand, the HOMO and LUMO diagrams of B-doped molecules Figures 4 and 5 have a good charge distribution within these doped molecules due to their very strong donor-acceptor nature linked to doping with boron. These results make our systems more interesting in electronic devices once more.

### 3.5 Optoelectronic properties

Optoelectronic parameters properties such as the refractive index (η), electric susceptibility (χ), the electric field (E), dielectric constant (ε), and polarization density (P) were calculated and listed in Table 5. These parameters values were computed using the formulas given in the literature [41, 42, 43, 44, 45]. We note that our computed results are very different when we move from undoped molecule to doped systems. Moreover, the E, P and η increases significantly and this difference is slightly between the uncorrelated to the DFT methods. Therefore, high values of the electric field E, the electric susceptibility χ, refractive index n and low value of dielectric constant Ɛ of doped systems find applications in optoelectronic materials. In fact, in renewable energy, photovoltaic and photonic devices.

**Table 5:** Dipole moment μ, average of the polarizability <α>, polarization density(P), the electric field (E), electric susceptibility (χ), dielectric constant (ε), and refractive index (n) of the molecules BEDT-TTF, 3B-, 2B- substituted C_10_H_8_S_8_ by employing the cc-pVDZ basis set.

Molecules parameters	$C_{10}H_{8}S_{8}$			$C_{7}B_{3}H_{5}S_{8}$			$C_{8}B_{2}H_{6}S_{8}$		
	B3LYP	wB97XD	RHF	B3LYP	wB97XD	RHF	B3LYP	wB97XD	RHF
**μ**(Cm) x10^−30^	4.70	5.17	3.25	7.51	6.46	4.34	12.75	11.1	8.27
**α**(C^2^.m^2^.J^−2^)x10^−41^	437.75	418.76	392.79	463.26	439.36	408.04	458.80	435.3	404.53
**V**(m^3^) x10^−30^	176.56	164.57	154.85	172.80	148.03	196.12	170.95	153.99	157.46
**E**(V.m^−1^) x10^9^	1.07	1.23	0.83	1.62	1.47	1.06	2.77	2.54	2.04
**P**(C.m^−2^) x10^−3^	26.62	31.41	20.99	43.46	43.64	22.13	74.55	72.08	52.51
**χ**_**e**_	2.81	2.88	2.86	3.03	3.35	2.36	3.04	3.2	2.9
**ε**_**r**_	3.81	3.88	3.86	4.03	4.35	3.36	4.04	4.2	3.9
**ε**x10^−12^	33.73	34.35	34.18	35.68	38.51	29.75	35.77	37.18	34.53
**n**	1.95	1.97	1.96	2.01	2.09	1.83	2.01	2.05	1.70

### 3.6 Thermodynamic properties

The various thermodynamic parameters presented in Table 6 such as Total electronic energy (Eelec), Zero vibrational point energy (ZPVE), Gibbs free energy (G), Thermal energy (E), Entropy (S), Enthalpy (H), constant volume calorific capacity (Cv) were calculated an ambient temperature of 298.15 K and a pressure of 1atm. Our results show that structures doped with boron have a greater total energy than the undoped molecule (C_10_H_8_S_8_). On the other hand, we observe a slight variation of the entropy, enthalpy and specific heat. The same difference is also observed when going from the HF to the two hybrid functional B3LYP and wB97XD. This is explained by the effect of correlation of the electrons taken into account in the both functional. Therefore, we are able to conclude that there is an influence of doping with boron on the entropy of molecular structures, which confirms that the charge dynamics of the doped molecules are higher than its original molecule at the same temperature. This result further demonstrates that these doped materials have a high chemical reactivity and a high thermal resistivity. A better agreement with work reported by Mveme et al [38].

**Table 6:** Total electronic energy (Eelec), Zero vibrational point energy (ZPVE), Gibbs free energy (G), Thermal energy (E), Entropy (S), Enthalpy (H), constant volume calorific capacity (Cv), of the molecules C_10_H_8_S_8_ (ET), C_7_B_3_H_5_S_8_ and C_8_B_2_H_6_S_8_ obtained using Hartree-Fock, B3LYP and wB97XD with CC-pVDZ basis set at T = 298.15K.

Molecules parameters	$C_{10}H_{8}S_{8}$			$C_{7}B_{3}H_{5}S_{8}$			$C_{8}B_{2}H_{6}S_{8}$		
	B3LYP	wB97XD	RHF	B3LYP	wB97XD	RHF	B3LYP	wB97XD	RHF
**Eelec**(a.u)	-3571.49	-3571.24	-3563.51	-3529.998	-3529.738	-3522.28	-3543.82	-3543.56	-3536.01
**ZPVE**(kcal/mol)	99.09	101.42	107.66	71.106	72.641	76.889	80.746	82.512	87.511
**Eo**(kcal/mol) x10^3^	-2241.05	-2240.874	-2236.03	-2215.035	-2214.371	-2210.19	-2223.703	-2223.538	-2218.798
**E**(kcal/mol) x10^3^	-2241.04	-2240.872	-2236.02	-2215.024	-2214.859	-2210.17	-2223.691	-2223.526	-2218.787
**H**(kcal/mol) x10^3^	-2241.04	-2240.871	-2236.02	-2215.023	-2214.859	-2210.178	-2223.690	-2223.435	-2218.789
**G**(kcal/mol) x10^3^	-2241.08	-2240.915	-2236.06	-2215.067	-2214.902	-2210.22	-2223.734	-2223.569	-2218.829
**E**thermal(kcal/mol)	111.11	113.122	118.87	83.028	84.366	88.127	92.643	94.212	98.704
**Cv**(cal/mol.k^1^)	69.38	67.718	64.68	69.38	68.15	65.44	69.132	67.774	64.952
**S**(cal/mol.k^1^)	148.47	145.81	144.18	146.32	145.22	142.03	146.930	146.69	143.047

## 4 Conclusion

In summary, the nonlinear optical, electronic, optoelectronic and thermodynamic properties of undoped and doped BEDT-TTF have been studied. Our goal was to investigate the effect or influence of doping with boron on the original compound. Our results obtained from the three methods used show that BEDT-TTF has weak NLO properties. However, substituted atom of carbon by boron show more interesting properties such as high first hyperpolarizability makes the molecule to find applications in NLO due to the π-electron conjugation. In fact, we presented for the first time the results on the substitution of C by B in the structure of BEDT-TTF. New systems obtained are good candidate to find application in renewable energy, emerging technologies. The HOMO-LUMO energy gap obtained lead us to believe that BEDT-TTF and its derivatives are good semiconductor materials that can be used in optoelectronic devices of telecommunications, in electronics, in LED, photonic materials and field effect transistor (FET). Finally, we are able to say that, these results reveal original electronic properties for our new materials, which can lead to interesting performances for organic photovoltaic, thus, opening the way to innovative and promising materials.

## Declarations

### Author contribution statement

G.F. OlingaMbala, M.T.Ottou Abe, Z. Ntieche, G.W.Ejuh, J.M.B.Ndjaka: Conceived and designed the experiments; Performed the experiments; Analyzed and interpreted the data; Contributed reagents, materials, analysis tools or data; Wrote the paper.

### Funding statement

This work was supported by Professor Geh Wilson Ejuh and their Mentor Emeritus Professor A.N. Singh through providing the GAUSSIAN code.

### Data availability statement

Data will be made available on request.

### Declaration of interests statement

The authors declare no conflict of interest.

### Additional information

No additional information is available for this paper.

## References

[1] Shakerzadeh E., Tahmasebi E., Biglari Z. (2016). A quantum chemical study on the remarkable nonlinear optical and electronic characteristics of boron nitride nanoclusters by complexation via lithium atom. J. Mol. Liq..

[2] Demiralp E., Dasgupta S., William A., Goddard (1995). Electron-Transfer boat-vibration mechanism for superconductivity in organic molecules based on BEDT-TTF. J. Am. Chem. Soc..

[3] Imamura Y., Ten-no S., Yonemitsu K., Tanimura Y. (1999). Structures and electronic phases of the bis(ethylenedithio) tetrathiafulvalene (BEDT-TTF) salts : a theoretical study based on *ab initio* molecular orbital methods. J. Chem. Phys..

[4] Demiralp E., Dasgupta S., William A., Goddard (1997). MSX force field and vibrational frequencies for BEDT-TTF (neutral and cation). J. Phys. Chem..

[5] Kozlov M.E., Pokhodnia K.I., Yurchenko A.A. (1987). The assignment of fundamental vibrations of BEDT-TTF and BEDT-TTF- d_8_. Spectrochim. Acta part A.

[6] Eldridge J.E., Homes C.C., Williams J.M., Kini A.M., Wang H.H. (1995). The assignment of the normal modes of the BEDT-TTF electron-donor molecule using the infrared and Raman spectra of several isotopic analogs. Spectrochim. Acta.

[7] Liu R., Zhou X., Kasmai H. (1997). Toward understanding the vibrational spectra of BEDT-TTF, a scaled density functional force field approach. Spectrochim. Acta, Part A.

[8] Flakina A.M., Chekhlov A.N., Luybovskaya R.N. (2004). New organic conductors based on TTF derivatives with polymeric isocyanuric acid anion. J. Phys. IV France.

[9] Wallis J.D., Griffiths J.P. (2005). Substituted BEDT-TTF derivatives: synthesis, chirality, properties and potential applications. J. Mater. Chem..

[10] Wang Q. (2015). A family of unsymmetrical hydroxyl-substituted BEDT-TTF donors: syntheses, structures and preliminary thin film studies. RSC Adv..

[11] Nakazawa Y., Yamashita S. (2012). Thermodynamic properties of k-(BEDT-TTF)_2_X Salts: electron correlations and superconductivity. Crystals.

[12] Girlando A. (2011). Charge sensitive vibrations and electr on-molecular vibration coupling in bis(ethylenedithio)-tetrathiafulvalene (BEDT-TTF). J. Phys. Chem. C.

[13] Pustogow A., Treptow K., Rohwer A., Saito Y., Sanz Alonso M., Löhle A., Schlueter J.A., Dressel M. (2019). Charge order in *β*’’-phase BEDT-TTF salts. Phys. Rev. B.

[14] Midoune A., Messaoudi A. (2021). DFT/TDDFT studies of the structural, electronic and NBO properties of some complexes with the tetrathiafulvalene-1,3-benzothiazole ligand. Inorg. Chim. Acta..

[15] Sundaraganesan N., Illakiamani S., Meganathan C., Joshua B.D. (2007). Vibrational spectroscopy investigation using *ab initio* and density functional theory analysis on the structure of 3-aminobenzotrifluoride. Spectrochim. Acta, Part A.

[16] Costa Stefane N., Freire Valder N., Caetano Ewerton W.S., Maia Francisco F., Barboza Carlos A., Fulco Umberto L., Eudenilson L. (2016). DFT calculations with van der Waals interactions of hydrated calcium carbonate crystals CaCO_3_·(H_2_O, 6H_2_O): structural, electronic, optical, and vibrational properties. J. Phys. Chem..

[17] Henriques J.M., Barboza C.A., Albuquerque E.L., Fulco U.L., Moreira E. (2015). Structural, optoelectronic, infrared and Raman spectra from firstprinciples calculations of γ-Cd(OH)_2_. J. Phys. Chem. Solid..

[18] Moreira E., Barboza C.A., Albuquerque E.L., Fulco U.L., Henriques J.M., Araújo A.I. (2015). Vibrational and thermodynamic properties of orthorhombic CaSnO_3_ from DFT and DFPT calculations. J. Phys. Chem. Solid..

[19] Frisch M.J. (2009). Gaussian 09, Revision A 1.

[20] Dennington R.T., Keith J.M. (2016). Gauss View, Version 6. SemichemInc, Shawnee Mission KS.

[21] Becke A.D. (1988). Density-functional exchange-energy approximation with correct asymptotic behavior. Phys. Rev. A.

[22] Lee Chengteh, Yang Weitao, Parr Robert G. (1988). Development of the Colle-Salvetti correlation-energy formula into a functional of the electron density. J. Phys. Rev. B.

[23] Guionneau P., Chasseau D., Judith Howard A.K., Dayc P. (2000). Neutral bis(ethylenedithio)tetrathiafulvalene at 100 K. ActaCryst.

[24] Allen F.H. (2002). The Cambridge structural database: a quarter of a million crystal structures and rising. Acta Cryst. B.

[25] Saji R.S., Prasana J.C., Muthu S., George J. (2020). Spectrochim. Acta Part A Mol. Spectrosc..

[26] Irikura K.K. (2002). THERMO. PL.

[27] Kolinzky P.V. (1992). New materials and their characterization for photonic device applications. Opt. Eng..

[28] Eaton D.F. (1991). Nonlinear optical materials. Science.

[29] Andraud C., Brotin T., Garcia C., Pelle F., Goldner P., Bigot B., Collet A. (1994). Theoretical and experimental investigations of the nonlinear optical properties of vanillin, polyenovanillin, and bisvanillin derivatives. J. Am. Chem. Soc..

[30] Geskin V.M., Lambert C., Bredas J.L. (2003). Origin of high second-and third-order nonlinear optical responseinammonio/boratodiphenylpolyenezwitterions: the remarkable role of polarizedaromatic groups. J. Am. Chem. Soc..

[31] Nakano M., Fujita H., Takahata M., Yamaguchi K. (2002). Theoretical study on second hyperpolarizabilities of phenylacetylenedendrimer: toward an understanding of structure-property relation in nloresponses of fractal antennadendrimers. J. Am. Chem. Soc..

[32] Sajan D., Joe H., Jayakumar V.S., Zaleski J. (2006). Structural and electronic contributions to hyperpolarizability in methyl phydroxybenzoate. J. Mol. Struct..

[33] Mveme C.D.D., Tchangnwa Nya F., W Ejuh G., Kamsi R.A.Y., Ndjaka J.M.B. (2020). Density functional theory study of optoelectronic, nonlinear optical, piezoelectric and thermodynamic properties of poly (3,4-ethylenedioxythiophene), poly(3,4-ethylenedioxyselenophene) and their derivative. Opt. Quant. Electron..

[34] Yossa Kamsi R.A., W Ejuh G., Nkounga P., Ndjaka J.M.B. (2020). Study of the molecular structure, electronic and chemical properties of Rubescin D molecule. Chin. J. Phys..

[35] Yossa Kamsi R.A., Ejuh G.W., Tchoffo F., Mkounga P., Ndjaka J.M.B. (2019). Electronic structure, spectroscopic (IR, Raman, UV-vis, NMR), optoelectronic, and NLO properties investigations of rubescin E (C31H36O7) molecule in gas phase and chloroform solution using ab initio and DFT. Methods.

[36] Midoune A., Messaoudi A. (2020). DFT/TD-DFT computational study of the tetrathiafulvalene-1,3-benzothiazole molecule to highlight its structural. electronic, vibrational and non-linear optical properties.

[37] Fonkem C.C., Ejuh G.W., Tchangnwa Nya F., Yossa Kamsi R.A., Tadjouteu Assatse Y., Ndjaka J.M.B. (2019). A density functional theory (DFT) study of the doping effect on 2-cyano-3-[4 (diphenylamino) phenyl] acrylic acid. Chin. J. Phys..

[38] Mveme C.D.D., Nya F.Tchangnwa., Ejuh G.W., Ndjaka J.M.B. (2021). A density functional theory (DFT) study of the doping effect on 4-[2-(2-N,N-dihydroxy amino thiophene) vinyl]benzenamine. SN applied Sciences.

[39] G.W Ejuh, M.T.Ottou Abe, T. Ghislain , J.M.B. Ndjaka, *Ab initio* and DFT studies on the donor–acceptor molecules1,2,3-trihydroxy-9,10-anthraquinone; 1(methylamino)anthraquinone; 2-phenyl quinoxaline and 2-(4-aminophenyl) quinoxaline (2018). Mater Focus 7: 37–44.

[40] Fankam J.B., Ejuh G.W., Tchangnwa Nya F., Ndjaka J.M.B. (2020). Theoretical investigation of the molecular structure, vibrational spectra, thermodynamic and nonlinear optical properties of 4, 5-dibromo-2, 7dinitro- fluorescein. Opt. Quant. Electron..

[41] Fankam Fankam J.B., Ejuh G.W., Tchangnwa Nya F., Ndjaka J.M.B. (2020). Study of electronic structure, optoelectronics, linear and nonlinear optical properties and chemical descriptors of dibromodinitrofluorescein isomers in gas phase and solvent media using ab initio and DFT methods. Chin. J. Phys..

[42] Kabé C., Tchangnwa Nya F., Ejuh G.W., Ndjaka J.M. (2020). Comparative study of optoelectronic, thermodynamic, linear and nonlinear optical properties of methyl phenalenyl doped to zinc and copper and their applications. J. Mater. Sci. Mater. Electron..

[43] Ejuh G.W., Ottou Abe M.T., Tchangwa Nya F., Ndjaka J.M.B. (2018). Prediction of electronic structure, dielectric and thermodynamical properties of flurbiprofen by density functional theory calculation. Karbala J. Mod. Sci..

[44] Ejuh G.W., Nouemo S., Tchangnwa Nya F., Ndjaka J.M.B. (2016). Computational determination of the electronic and nonlinear optical properties of the molecules 2-(4-aminophenyl) quinoline, 4-(4-aminophenyl) quinoline, anthracene, anthraquinone and phenanthrene. Mater. Lett..

[45] Tchangnwa Nya F., Ejuh G.W., Ndjaka J.M.B. (2017). Theoretical study of optoelectronic and thermodynamic properties of molecule 4-[2-(2-N,N-dihydroxy amino thiophene)vinyl] benzanamine: influence of hydroxyl position. Mater. Lett..

